# A clinical prediction model for low psoas muscle radiodensity in adults with severe obesity: development and internal validation

**DOI:** 10.3389/fnut.2026.1864098

**Published:** 2026-07-13

**Authors:** Eunhye Seo, Mi Kyung Kim, Suh Youngsung, Yu-Shan Hsieh, Seung-Wan Ryu

**Affiliations:** 1 Department of Nursing, College of Nursing, Keimyung University, Daegu, Republic of Korea; 2 Department of Internal Medicine, Keimyung University School of Medicine, Daegu, Republic of Korea; 3 Department of Family Medicine, Keimyung University Dongsan Medical Centre, Daegu, Republic of Korea; 4 School of Nursing, National Taipei University of Nursing and Health Sciences, Taipei, Taiwan; 5 Department of Surgery, Keimyung University Dongsan Medical Centre, Daegu, Republic of Korea

**Keywords:** age, albumin, body mass index, phase angle, psoas muscle radiodensity, severe obesity

## Abstract

**Introduction:**

Low psoas muscle radiodensity (PMD) on computed tomography (CT) indicates impaired muscle quality and is widely used as an operational marker of myosteatosis in individuals with obesity. In this study, we aimed to develop and internally validate a clinically accessible prediction model for low PMD in adults with severe obesity.

**Methods:**

This retrospective study included 150 adults with severe obesity, defined as body mass index (BMI) ≥ 35 kg/m^2^, who underwent CT evaluation as candidates for metabolic and bariatric surgery at our university hospital between January 2019 and May 2025. PMD was quantified on CT, and low PMD was defined as the lowest quartile [cutoff: ≤40.8 Hounsfield units (HU)]. A prediction model incorporating age, BMI, serum albumin, and bioelectrical impedance analysis-derived phase angle (PhA) was developed. Model performance was assessed using discrimination and accuracy metrics, with internal validation by bootstrapping.

**Results:**

The mean age, BMI, and PMD were 37.0 ± 10.6 years, 40.30 ± 8.05 kg/m^2^, and 45.90 ± 8.76 HU, respectively. PhA provided incremental explanatory value for PMD (Δ*R*^2^ = 0.048, *p* = 0.001). In multivariable logistic regression analysis, age and BMI were independently associated with low PMD, whereas albumin and PhA were not significantly associated with low PMD; however, their incremental contribution was assessed based on predictive performance. The integrated model demonstrated improved discrimination compared with the age–BMI model [area under the curve (AUC) = 0.841 vs. 0.829]. At an optimal cutoff of 0.38, the sensitivity was 67.5%, specificity was 85.5%, and optimism-corrected AUC was 0.821.

**Discussion:**

This clinically accessible model demonstrated good discrimination and high specificity, supporting clinical risk stratification in settings where CT is limited.

## 1 Introduction

The global prevalence of obesity, defined as a body mass index (BMI) of ≥30 kg/m^2^, has continued to rise between 1990 and 2022, from 8.8 to 18.5% in women and 4.8 to 14.0% in men (1). Concurrently, the increasing prevalence of severe obesity has been characterized as a high-demand epidemic associated with substantial clinical and health-system burden (2). As eligibility for intensive obesity treatments expands, pragmatic tools for stratifying patients are increasingly needed to address heterogeneity in treatment responses, regarding weight loss and accompanying body composition changes (3, 4). One clinically salient dimension of this heterogeneity is variability in muscle-related outcomes, including fat-free mass loss accompanying weight reduction (5, 6).

Although obesity management encompasses lifestyle intervention, pharmacotherapy, and surgery, treatment success is not fully characterized by the magnitude of weight loss alone, because weight reduction commonly involves loss of fat-free mass, including skeletal muscle (6, 7). A systematic review and meta-analysis reported clinically meaningful losses in lean body mass and fat-free mass within 12 months after bariatric surgery, with a substantial proportion occurring early after surgery (8). Glucagon-like peptide-1–based anti-obesity therapies have been reported to be accompanied by proportional reductions in lean mass, with heterogeneity observed across trials (9). Accordingly, “high-quality weight loss” has been defined as weight loss in which relatively greater fat loss than skeletal muscle loss occurs, reflecting the central roles of skeletal muscle in energy expenditure, glucose homeostasis, mobility, and strength (5, 7, 10). Beyond preserving muscle quantity, muscle quality is increasingly being recognized as relevant to metabolic health and functional outcomes (11).

Furthermore, skeletal muscle quantity may appear relatively preserved in obesity, while muscle quality is impaired due to ectopic lipid infiltration of the muscle tissue (12, 13). This condition, termed myosteatosis, is most commonly quantified on computed tomography (CT) as reduced muscle radiodensity using Hounsfield units (HU) (14). CT-derived myosteatosis has been associated with dysglycemia, insulin resistance, type 2 diabetes, and inflammation, independent of general obesity and visceral fat, and has also been linked to bariatric surgery response in severe obesity (15, 16). However, routine CT-based assessment is constrained by availability, radiation exposure, and analytic resources, and definitions and thresholds vary substantially across studies (17, 18).

Given these constraints, clinically accessible approaches are needed to identify individuals at increased risk of impaired muscle quality when CT-based quantification is unavailable. Candidate measures reflecting aging-related tissue remodeling, obesity severity, systemic nutritional and inflammatory status, and cellular integrity and hydration may plausibly relate to muscle lipid infiltration and radiodensity (17, 19–21), which decline with age (22). BMI, while an imperfect proxy for body composition, remains a pragmatic indicator of obesity severity and can contribute to baseline risk stratification when interpreted alongside complementary markers (23). Serum albumin, an inexpensive biomarker reflecting nutritional and systemic inflammatory status, has been evaluated in clinical cohorts (19). Finally, bioelectrical impedance analysis (BIA)-derived phase angle (PhA), which has been investigated as an accessible indicator of muscle health, has shown associations with imaging-based markers of muscle quality, including CT-derived skeletal muscle radiodensity (20, 24).

Prediction models for low muscle radiodensity have been developed largely in oncology cohorts, including nomograms reported among populations with gastric and colorectal cancers (25, 26), limiting direct generalizability to adults with a BMI ≥ 35 kg/m^2^. Therefore, we aimed to develop and internally validate a pragmatic model based on routinely available clinical variables. This aims to identify adults with BMI ≥ 35 kg/m^2^ who are at higher risk of low psoas muscle radiodensity on CT, thereby supporting clinical triage for impaired muscle quality when CT-based quantification is unavailable.

## 2 Materials and methods

### 2.1 Study participants

All adults aged ≥18 years evaluated for bariatric surgery at the Obesity and Metabolic Center of Keimyung University Dongsan Hospital between January 2019 and May 2025 were systematically screened (*n* = 225). Among them, patients with BMI ≥ 35 kg/m^2^ who had undergone abdominopelvic CT as part of a standardized preoperative workup were considered potentially eligible. Consecutive eligible patients were included.

Participants were excluded if they met any of the following criteria: (i) a history of previous bariatric or upper gastrointestinal surgery (*n* = 2); (ii) significant medical conditions that could independently influence muscle quality (*n* = 62); (iii) no usable CT images for PMD quantification (*n* = 3); (iv) unusable or missing key clinical variables, including age, BMI, serum albumin, and BIA-derived PhA measured within ± 3 days of the CT examination (*n* = 8).

The requirement for informed consent was waived due to the retrospective design and use of de-identified data, and the study design was approved by the appropriate ethics review board.

### 2.2 BIA analysis and blood test

After an overnight fast (≥8 h), BIA was performed using a multifrequency analyzer (InBody 770; InBody Co., Seoul, Korea), with participants in the standing position and all metallic objects removed. Whole-body PhA at 50 kHz was obtained directly from the device output. Routine blood tests were performed after the same fasting period.

### 2.3 CT image analysis

Abdominal CT images were acquired using a 128-detector CT scanner (SOMATOM Definition Flash; Siemens, Munich, Germany) as part of a standardized preoperative protocol for patients scheduled for bariatric surgery. All examinations were performed as contrast-enhanced CT in the portal venous phase according to institutional protocol.

A single axial image at the third lumbar vertebra (L3) level was selected for analysis. The bilateral psoas muscles were segmented using Slice-O-Matic software (version 4.3; TomoVision, Montreal, Canada), and PMD was calculated as the mean attenuation (HU) across the segmented psoas muscles.

All image analyses were performed by a trained investigator blinded to participants’ clinical and laboratory information. Intraobserver and interobserver reliability were assessed using repeated measurements from 30 randomly selected participants and an independent second investigator, respectively (16).

### 2.4 Definition of low PMD

Low PMD was defined as the lowest quartile (Q1) of PMD in the study population, corresponding to a cutoff value of 40.8 HU. This psoas-specific threshold was adopted because PMD is reportedly systematically higher than the radiodensity of whole L3-level skeletal muscle (18). Participants were categorized as low (Q1) or normal (Q2–Q4) PMD. Skeletal muscle index (SMI) was calculated by normalizing the cross-sectional skeletal muscle area at the L3 vertebral level to height squared (cm^2^/m^2^).

### 2.5 Statistical analysis

Statistical analyses were performed using jamovi (version 2.6.44; The jamovi project, Sydney, Australia) and R software (version 4.4.1; R Foundation for Statistical Computing, Vienna, Austria). Participants with missing data on key variables were excluded, and all analyses were conducted using a complete-case approach.

Continuous variables are presented as mean ± standard deviation and categorical variables as counts. Between-group comparisons were performed using Student’s *t*-test or the Mann–Whitney U test for continuous variables and the χ^2^ test or Fisher’s exact test for categorical variables.

Associations between clinical variables and PMD were examined using correlation and linear regression analyses. Independent factors associated with low PMD (binary outcome) were assessed using multivariable logistic regression analysis.

Diagnostic performance of individual predictors and combined models was evaluated using receiver operating characteristic (ROC) curves and area under the curve (AUC). An optimism-corrected AUC was calculated using R software to account for potential overfitting and estimate model performance in new populations, consistent with TRIPOD reporting guidelines. Internal validity was evaluated using bootstrap resampling with 1,000 iterations.

The optimal probability cutoff was determined using the Youden index, and sensitivity and specificity were calculated. Parallel mediation analysis was conducted with 5,000 bootstrap resamples. All tests were two-sided, and *p* < 0.05 was considered statistically significant.

Decision curve analysis (DCA) was performed to evaluate the clinical utility of the prediction models across a range of threshold probabilities, quantifying the net benefit of each model compared with the treat all and treat none strategies.

## 3 Results

### 3.1 Participant characteristics

Table 1 shows the characteristics of the included 150 patients. Their mean age, BMI, and PMD were 37.0 ± 10.6 years, 40.30 ± 8.05 kg/m^2^, and 45.90 ± 8.76 HU, respectively. Participants were classified as low (Q1, *n* = 40) or normal (Q2–4, *n* = 110) PMD, with significantly lower PMD in the low PMD group (*p* < 0.001). Age, BMI, body fat percentage, and waist and hip circumferences were higher, whereas PhA was lower in the low PMD group than in the normal PMD group (all *p* ≤ 0.005).

**Table 1:** Participant characteristics.

Variables		Total (*n* = 150)	Low PMD (Q1, *n* = 40)	Normal PMD (Q2–4, *n* = 110)	*p*-value
PMD, HU		45.90 ± 8.76	35.27 ± 6.46	49.77 ± 5.79	<0.001
Sex	Female	102 (68.0)	29 (28.4)	73 (71.5)	0.435
	Male	48 (32.0)	11 (22.9)	37 (77.0)	
Age, years		37.0 ± 10.6	41.37 ± 11.49	35.45 ± 9.81	0.004 ^**a**^
BMI, kg/m^2^		40.30 ± 8.05	45.15 ± 10.54	38.54 ± 6.08	<0.001 ^**a**^
WC, cm		119.71 ± 18.92	126.67 ± 18.95	115.70 ± 18.07	0.004 ^**a**^
HC, cm		122.34 ± 15.70	128.96 ± 20.51	119.28 ± 20.51	0.004 ^**a**^
Body fat, %		47.00 ± 5.74	49.31 ± 6.16	46.13 ± 5.16	0.004 ^**a**^
SMI		8.86 ± 1.25	9.21 ± 1.62	8.73 ± 1.07	0.090 ^a^
PhA, degree (°)		5.70 ± 0.64	5.45 ± 0.64	5.79 ± 0.62	0.005
Total protein, g/dL		7.10 ± 0.42	7.06 ± 0.43	7.11 ± 0.42	0.474
Albumin, g/dL		4.44 ± 0.37	4.30 ± 0.51	4.49 ± 0.29	0.012 ^**a**^
Iron, μg/dL		82.21 ± 29.40	71.60 ± 30.54	86.04 ± 28.15	0.004 ^**a**^
ln(hsCRP)		1.16 ± 0.93	1.39 ± 1.09	1.08 ± 0.86	0.045
LDH, U/dL		511.67 ± 135.23	550.75 ± 152.34	501.38 ± 125.65	0.044 ^**a**^
T3, ng/dL		144.21 ± 21.3	137.18 ± 20.77	146.99 ± 21.01	0.011
T4, ng/dL		1.30 ± 1.88	1.11 ± 0.34	1.36 ± 2.18	0.438 ^a^
TSH, μIU/dL		3.02 ± 4.64	4.88 ± 8.55	2.34 ± 1.30	0.033 ^**a**^
Glucose, mg/dL		114.21 ± 35.60	115.20 ± 29.82	113.55 ± 37.57	0.143 ^a^
HOMA IR		4.87 ± 3.53	5.35 ± 4.27	4.68 ± 3.23	0.509 ^a^
HbA1C, %		6.27 ± 1.27	6.19 ± 1.05	6.30 ± 1.34	0.603 ^a^
Total cholesterol, mg/dL		185.12 ± 54.90	183.87 ± 40.48	185.27 ± 59.38	0.726 ^a^
LDL cholesterol, mg/dL		119.18 ± 35.30	119.05 ± 36.94	118.44 ± 34.92	0.926
HDL cholesterol, mg/dL		44.51 ± 11.39	45.40 ± 11.28	44.10 ± 11.32	0.537 ^a^
Triglyceride, mg/dL		185.00 ± 359.01	151.62 ± 131.87	196.69 ± 411.31	0.070 ^a^
Comorbidities					
Type 2 diabetes	Yes	59 (39.3)	18 (12.0)	41 (27.3)	0.370
	No	91 (60.7)	22 (14.7)	69 (46.0)	
Hypertension	Yes	60 (40.0)	20 (13.3)	40 (26.7)	0.132
	No	90 (60.0)	20 (13.3)	70 (46.7)	
Hyperlipidemia	Yes	77 (51.3)	18 (12.0)	59 (39.3)	0.349
	No	73 (48.7)	22 (14.7)	51 (34.0)	
Disease duration, years					
Type 2 diabetes		1.55 ± 4.08	1.20 ± 3.02	1.68 ± 4.41	0.520 ^a^
Hypertension		1.50 ± 3.38	2.30 ± 3.84	1.21 ± 3.17	0.075 ^a^
Hyperlipidemia		0.97 ± 2.79	0.87 ± 2.29	1.01 ± 2.96	0.777 ^a^

Data are presented as mean ± SD or number (%). *p*-values were calculated using the independent *t*-test or the Mann–Whitney U test (indicated by ^a^) for continuous variables, and the Chi-square test for categorical variables. Due to its skewed distribution, hsCRP was log-transformed and analyzed as ln(hsCRP) for statistical analysis. PMD, psoas muscle density; HU, Hounsfield units; BMI, body mass index; SMI, skeletal muscle index; PhA, phase angle; ln(hsCRP), natural logarithm of high-sensitivity C-reactive protein; LDH, lactate dehydrogenase; T3, triiodothyronine; T4, thyroxine; TSH, thyroid-stimulating hormone; HOMA-IR, homeostatic model assessment for insulin resistance; HbA1C, glycated hemoglobin; LDL, low-density lipoprotein; HDL, high-density lipoprotein; WC, waist circumference; HC, hip circumference; SD, standard deviation.

Regarding biochemical markers, the low PMD group showed lower albumin, iron, and thyronine (T3), and higher natural logarithm of high-sensitivity C-reactive protein [ln(hsCRP)], lactate dehydrogenase (LDH), and thyroid-stimulating hormone (TSH) (all *p* < 0.05). No significant between-group differences were observed in thyroxine (T4), glucose metabolism markers, lipid profiles, total iron-binding capacity, ferritin, vitamin B12, vitamin D, and folate levels. Furthermore, the prevalence and duration of comorbidities, including type 2 diabetes, hypertension, and hyperlipidemia, were comparable between groups (Table 1).

### 3.2 Association of clinical variables with PMD

In Supplementary Table 1, unadjusted analysis showed that PMD was positively correlated with PhA, albumin, iron, and T3, and negatively correlated with BMI and body fat percentage (all *p* < 0.01). After adjusting for age and sex, PMD also showed significant negative correlations with ln(hsCRP), LDH, and TSH (all *p* < 0.05).

Linear regression analysis for PMD is presented in Table 2. In the final model, the addition of PhA significantly increased the explanatory power of the model (Δ*R*^2^ = 0.048, *p* = 0.001), accounting for 34.8% of the total variance (adjusted *R*^2^ = 0.348). After adjusting for all covariates, PhA remained the strongest positive predictor of PMD (*B* = 3.700, *p* = 0.001). Additionally, age (*B* = −0.291, *p* < 0.001), BMI (*B* = −0.328, *p* = 0.008), and albumin (*B* = 3.469, *p* = 0.040) were significant independent predictors of PMD.

**Table 2:** Hierarchical linear regression analysis of independent predictors of PMD.

Variables	Model 1			Model 2			Model 3		
	B (95% CI)	*β*	*p*-value	B (95% CI)	*β*	*p*-value	B (95% CI)	*β*	*p*-value
Age	−0.286 (−0.413, −0.159)	−0.347	<0.001	−0.326 (−0.459, −0.193)	−0.395	<0.001	−0.291 (−0.421, −0.161)	−0.353	<0.001
Sex	−1.101 (−3.966, 1.763)	−0.126	0.449	0.283 (−3.225, 3.793)	0.032	0.873	1.229 (−2.208, 4.665)	0.140	0.480
BMI				−0.269 (−0.517, −0.022)	−0.248	0.033	−0.328 (−0.569, −0.086)	−0.302	0.008
Body fat				−0.116 (−0.479, 0.246)	−0.076	0.528	−0.157 (−0.523, 0.208)	−0.103	0.396
Albumin				4.006 (0.601, 7.412)	0.171	0.021	3.469 (0.165, 6.774)	0.148	0.040
Iron				0.033 (−0.013, 0.079)	0.110	0.162	0.020 (−0.024, 0.066)	0.069	0.364
ln(hsCRP)				0.230 (−1.427, 1.889)	0.024	0.784	0.013 (−1.593, 1.169)	0.001	0.987
LDH				−0.005 (−0.015, 0.003)	−0.092	0.224	−0.006 (−0.016, 0.002)	−0.107	0.144
T3				0.038 (−0.024, 0.100)	0.093	0.227	0.028 (−0.032, 0.088)	0.069	0.356
TSH				−0.173 (−0.444, 0.098)	−0.092	0.209	−0.168 (−0.430, 0.093)	−0.089	0.206
PhA							3.700 (1.499, 5.902)	0.275	0.001
Model summary									
*R* ^2^	0.129				0.347		0.396		
Adjusted *R*^2^	0.117				0.301		0.348		
△*R*^2^	–				0.218		0.048		
p for △*R*^2^	–				<0.001		0.001		
Max VIF	1.50				2.13		2.13		

*p*-values were calculated using hierarchical linear regression analysis with PMD as the dependent variable. CI, confidence interval; PMD, psoas muscle density; BMI, body mass index; PhA, phase angle; ln(hsCRP), natural logarithm of high-sensitivity C-reactive protein; LDH, lactate dehydrogenase; T3, triiodothyronine; TSH, thyroid-stimulating hormone; VIF, variance inflation factor.

### 3.3 Multivariable logistic prediction model for low PMD

A multivariable logistic regression model was developed to predict the probability of low PMD. The final model incorporated age, BMI, serum albumin, and PhA, and the corresponding model coefficients are presented in Table 3. In the final model, higher age and BMI were associated with higher predicted odds of low PMD, whereas albumin and PhA showed inverse associations. Albumin and PhA were retained in the final prediction model based on their independent associations with PMD and biological relevance to skeletal muscle quality.

**Table 3:** Multivariable logistic regression model coefficients for low PMD.

Variables	β coefficient	OR (95% CI)
Intercept	−4.871	NA
Age	0.107	1.112 (1.057, 1.171)
BMI	0.172	1.188 (1.104, 1.288)
Albumin	−0.880	0.414 (0.138, 1.240)
PhA	−0.623	0.536 (0.257, 1.120)

*p*-values were calculated using multivariate logistic regression analysis. Low PMD was defined as the lowest quartile (Q1) of PMD. PMD, psoas muscle density; BMI, body mass index; PhA, phase angle; OR, odds ratio; CI, confidence interval; NA, not applicable.

### 3.4 Predictive performance of the models for predicting low PMD

**Figure 1:**
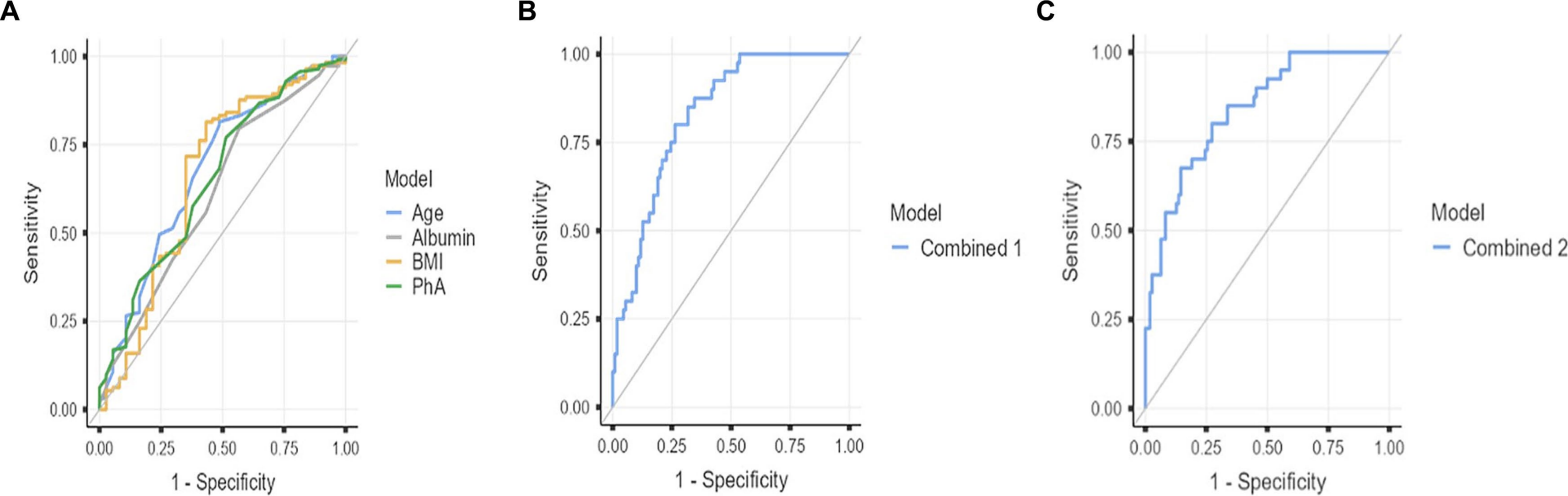
Diagnostic performance of individual variables and combined models for predicting low PMD. **(A)** ROC curves of individual predictors [age, body mass index (BMI), serum albumin, and phase angle (PhA)]. **(B)** ROC curve of the Combined 1 model including age and BMI. **(C)** ROC curve of the Combined 2 model including age, BMI, albumin, and PhA. *p*-values were calculated using ROC curve analysis. Combined 1 includes age and BMI. Combined 2 includes age, BMI, albumin, and PhA. Cutoff values were determined using the Youden index. PMD, psoas muscle density; BMI, body mass index; PhA, phase angle; AUC, area under the curve; CI, confidence interval.

To evaluate the predictive utility of the identified factors, ROC curve analysis was performed for individual variables and combined models (Table 4 and Figure 1). Among the individual predictors, age showed the highest diagnostic performance with an AUC of 0.678 [95% confidence interval (CI) = 0.574–0.782, *p* < 0.001] followed by PhA [AUC = 0.654 (95% CI = 0.549–0.758, *p* = 0.004)], BMI (AUC = 0.615, *p* = 0.006), and albumin (AUC = 0.615, *p* = 0.035).

**Table 4:** Diagnostic performance of individual variables and combined models for predicting low PMD.

Variables	AUC (95% CI)	*p*-value	Cut-off	Sensitivity, %	Specificity, %
Age	0.678 (0.574, 0.782)	<0.001	43.50	81.42	51.35
Albumin	0.615 (0.508, 0.722)	0.035	4.25	79.65	43.24
BMI	0.615 (0.547, 0.775)	0.006	43.29	81.42	56.76
PhA	0.654 (0.549, 0.758)	0.004	5.35	76.99	48.65
Combined 1	0.829 (0.764, 0.895)	<0.001	0.26	80.00	73.64
Combined 2	0.841 (0.773, 0.909)	<0.001	0.38	67.50	85.45

*p*-values and 95% CIs were calculated using receiver operating characteristic (ROC) curve analysis. Low PMD was treated as the dependent variable, and age, body mass index (BMI), serum albumin, and phase angle (PhA) were included as predictors. Combined 1 included age and BMI, and Combined 2 included age, BMI, albumin, and PhA. Optimal cutoff values were determined using the Youden index, and corresponding sensitivity and specificity were calculated. PMD, psoas muscle radiodensity; BMI, body mass index; PhA, phase angle; AUC, area under the curve; CI, confidence interval.

Diagnostic accuracy improved when multiple variables were integrated. The AUC for the Combined 1 model (age and BMI) was 0.829 (95% CI = 0.764–0.895, *p* < 0.001). The final Combined 2 model (age, BMI, albumin, and PhA) demonstrated the highest predictive performance with an AUC of 0.841 (95% CI = 0.773–0.909, *p* < 0.001).

**Figure 2:**
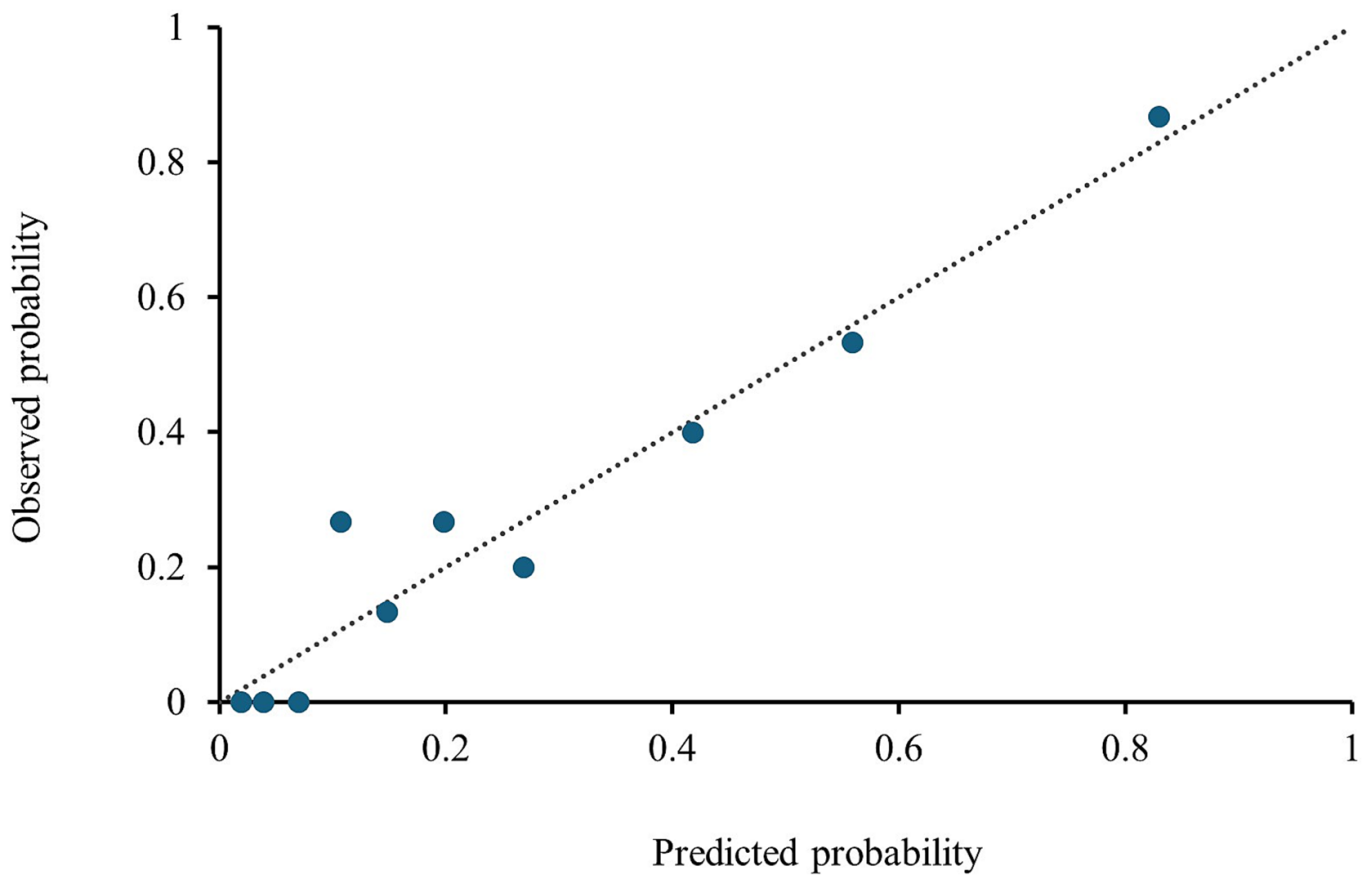
Calibration plot of the prediction model for low psoas muscle radiodensity (PMD). Observed event rates are plotted against mean predicted probabilities across deciles of predicted risk. The dashed diagonal line represents perfect calibration. The calibration slope was 1.00, the calibration intercept was approximately 0, and the Brier score was 0.135, indicating acceptable apparent calibration and overall prediction accuracy. PMD, psoas muscle radiodensity.

Internal validation using 1,000 bootstrap resamples showed an optimism of 0.019 for the Combined 2 model, resulting in an optimism-corrected AUC of 0.821. The Combined 1 model yielded an optimism-corrected AUC of 0.822. This reduction between apparent and corrected performance suggests minimal overfitting. Calibration analysis demonstrated acceptable agreement between predicted and observed probabilities of low PMD across deciles of predicted risk, with minor deviations at the extremes of predicted probability (Figure 2). Quantitative calibration assessment showed a calibration slope of 1.00 and a calibration intercept close to 0, indicating good apparent calibration. The Brier score was 0.135, suggesting acceptable overall prediction accuracy.

At the optimal cutoff value of 0.38, the Combined 2 model achieved a sensitivity of 67.50% and a specificity of 85.45%, compared with the Combined 1 model (sensitivity: 80.00%, specificity: 73.64%) (Table 4 and Figure 1).

### 3.5 Model performance and clinical utility

Decision curve analysis demonstrated that both the Combined 1 and Combined 2 models provided positive net benefit over a range of clinically relevant threshold probabilities (approximately 0.10–0.50), outperforming both the “treat-all” and “treat-none” strategies (Supplementary Figure 1). The Combined 2 model showed net benefit that was comparable to or marginally superior to the Combined 1 model across this range suggesting modest incremental clinical utility of integrating albumin and PhA alongside age and BMI for identifying individuals at risk of low PMD.

### 3.6 Mediation analysis of associations between PhA, BMI, and PMD

To further contextualize these findings, exploratory analyses were conducted. The results of the parallel mediation model with 5,000 bootstrap samples, examining potential indirect effects of demographic factors on PMD. PhA showed a significant negative indirect effect in the association between sex and PMD (*B* = −2.303; 95% CI = −4.058 to −0.953, *p* = 0.002), whereas BMI showed a significant positive indirect effect in the association between age and PMD (B = 0.078; 95% CI = 0.032–0.137, *p* = 0.004). No significant indirect effects were observed through albumin for either age or sex. For direct effects, age remained significantly negatively associated with PMD (*B* = −0.332, *p* < 0.001), while the direct effect of sex on PMD was not significant. Accordingly, age showed a significant total effect on PMD (*B* = −0.286, *p* < 0.001).

## 4 Discussion

This study developed and validated a clinical prediction model for myosteatosis, defined as low PMD, specifically in patients with severe obesity, based on age, BMI, albumin, and PhA. Through internal validation, the integrated model demonstrated robust performance (AUC = 0.841; sensitivity: 67.50%; specificity: 85.45%) and an optimism-corrected AUC of 0.821. Notably, although the age-BMI model yielded a comparable corrected AUC (0.822), the final integrated model provides superior clinical utility by improving specificity (85.45% vs. 73.64%), thereby reducing false-positive identifications in clinical triage (Figure 1). These findings support the robustness and potential clinical applicability of the integrated model in severe obesity. In addition to optimism-corrected discrimination, the model demonstrated acceptable apparent calibration and overall prediction accuracy, supporting the internal stability of the proposed model despite the relatively limited sample size.

In this study, low PMD was operationally defined as the lowest quartile of PMD, with a cutoff value of 40.8 HU. For BMI ≥ 25 kg/m^2^, a standardized mean difference-based myosteatosis threshold of <33 HU is commonly used (17, 18). However, PMD values are systematically higher than whole L3 skeletal muscle radiodensity, with a reported mean difference of approximately 1.7 HU (42.0 ± 8.4 vs. 32.3 ± 9.5 HU) (27). Consequently, psoas-based classification may require higher cutoff values than those derived from whole L3 muscle measurements (27). Accordingly, our lowest quartile-based cutoff can be interpreted as an operational threshold reflecting PMD. Consistently, in our study, the mean PMD in the low PMD group (35.27 ± 6.46 HU) was lower than reported values in general populations (42.0 ± 8.4 HU) (27), likely reflecting the higher obesity severity of our study population (BMI ≥ 35 kg/m^2^) compared with previously reported cohorts including individuals with BMI ≥ 25 kg/m^2^, thereby supporting the population-specific nature of the derived cutoff (6).

The superior performance of the integrated model compared with single-variable models suggests that myosteatosis in patients with severe obesity cannot be sufficiently explained by aging or BMI-defined adiposity alone, implying substantial heterogeneity in muscle quality (19). Body composition-based measures have been associated with metabolic outcomes after MBS, whereas BMI alone provides limited information regarding muscle phenotype and metabolic heterogeneity (20, 27). Skeletal muscle quantity and quality represent related but distinct dimensions of muscle health. Consistent with this concept, although SMI did not differ significantly between groups, PMD showed significant differences, suggesting that deterioration in muscle quality may occur independently of muscle quantity in severe obesity (22).

In our data, the addition of PhA and albumin improved model discrimination primarily by increasing specificity, indicating an incremental role in refining classification rather than serving as dominant determinants. Although these variables were not statistically significant independent predictors in multivariable logistic regression (Table 3), their inclusion improved model specificity in ROC analysis, suggesting that they contribute complementary information that reduces false-positive identification of low PMD (Table 4 and Figure 1).

To explore the biological basis of this incremental improvement, exploratory mediation analysis was conducted. BMI showed a significant indirect effect in the association between age and PMD, while PhA showed a significant indirect effect in the association between sex and PMD. These findings may suggest that the observed associations involve multiple pathways rather than a single adiposity-driven mechanism. Consistent with prior evidence linking aging to reduced muscle radiodensity independent of adiposity (22), our analysis suggests that age may be associated with PMD through both direct and indirect relationships involving BMI. In contrast, sex differences in PMD were not observed as direct effects but may be partly reflected in associations with PhA. These findings should be interpreted cautiously, as the cross-sectional design precludes causal inference. Albumin did not function as a significant mediator, supporting the interpretation that albumin reflects systemic vulnerability rather than directly explaining variation in muscle quality (25). Taken together, these findings suggest that sex differences in muscle radiodensity in severe obesity may partly reflect differences in cellular-level muscle properties rather than adiposity alone (23, 24). These pathway-specific effects support the integration of complementary clinical markers when identifying individuals at risk for low PMD.

Notably, in this relatively young study population (mean age: 37 ± 10.6 years), low PMD was not accompanied by differences in insulin resistance, glycated hemoglobin, or type 2 diabetes prevalence (Table 1). This finding suggests that ectopic fat infiltration within skeletal muscle may precede overt metabolic derangements and represent an early stage of muscle-quality impairment in severe obesity (10). Consistent with longitudinal evidence linking CT-derived muscle fat infiltration in midlife to subsequent cardiometabolic disease (26, 28), these observations support the potential role of low PMD as an early risk marker. As CT-based quantification is not routinely feasible in all clinical settings, identifying at-risk individuals using clinically accessible indicators may enable earlier risk stratification before overt metabolic deterioration becomes apparent. Prospective studies are warranted to determine whether low PMD and the proposed model predict incident cardiometabolic comorbidities and differential responses to MBS, thereby clarifying their role in risk stratification and personalized perioperative management.

### 4.1 Limitations

The lowest quartile-based PMD threshold reflects psoas radiodensity characteristics but requires external validation and standardization. PMD was assessed using contrast-enhanced CT obtained during routine preoperative evaluation. Therefore, the proposed model should be interpreted as a complementary screening approach for individuals already undergoing CT assessment, rather than as a recommendation for universal CT-based screening. In addition, differences in CT acquisition protocols and contrast enhancement may affect absolute PMD values across studies.

## 5 Conclusion

This study developed and internally validated a clinically accessible prediction model for low PMD in candidates for MBS. The integrated model improved specificity compared with conventional indicators, supporting the concept that muscle quality impairment reflects multidimensional biological processes rather than adiposity alone. As low PMD may represent an early stage of muscle-quality deterioration, this model may enable risk stratification using routinely available clinical measures when CT assessment is unavailable.

## Data Availability

The raw data supporting the conclusions of this article will be made available by the authors, without undue reservation.

## Supplementary material

Supplementary Figure 1 Decision curve analysis for the prediction models of low psoas muscle radiodensity (PMD). The y-axis represents net benefit and the x-axis represents the threshold probability. The solid line represents the Combined 2 model (age, BMI, albumin, and phase angle); the dashed line represents the Combined 1 model (age and BMI); the dotted line represents the “treat all” strategy; and the dash-dotted line represents the “treat none” strategy (net benefit = 0). Both models demonstrated positive net benefit across a range of threshold probabilities from approximately 0.10–0.50, supporting their clinical utility for risk stratification when CT-based assessment is unavailable.
