# Supplementary figures and images for: A clinical prediction model for low psoas muscle radiodensity in adults with severe obesity: development and internal validation
Source: Front Nutr. 2026 Jul 13;13:1864098. doi: 10.3389/fnut.2026.1864098 (PMC13403797; doi:10.3389/fnut.2026.1864098)

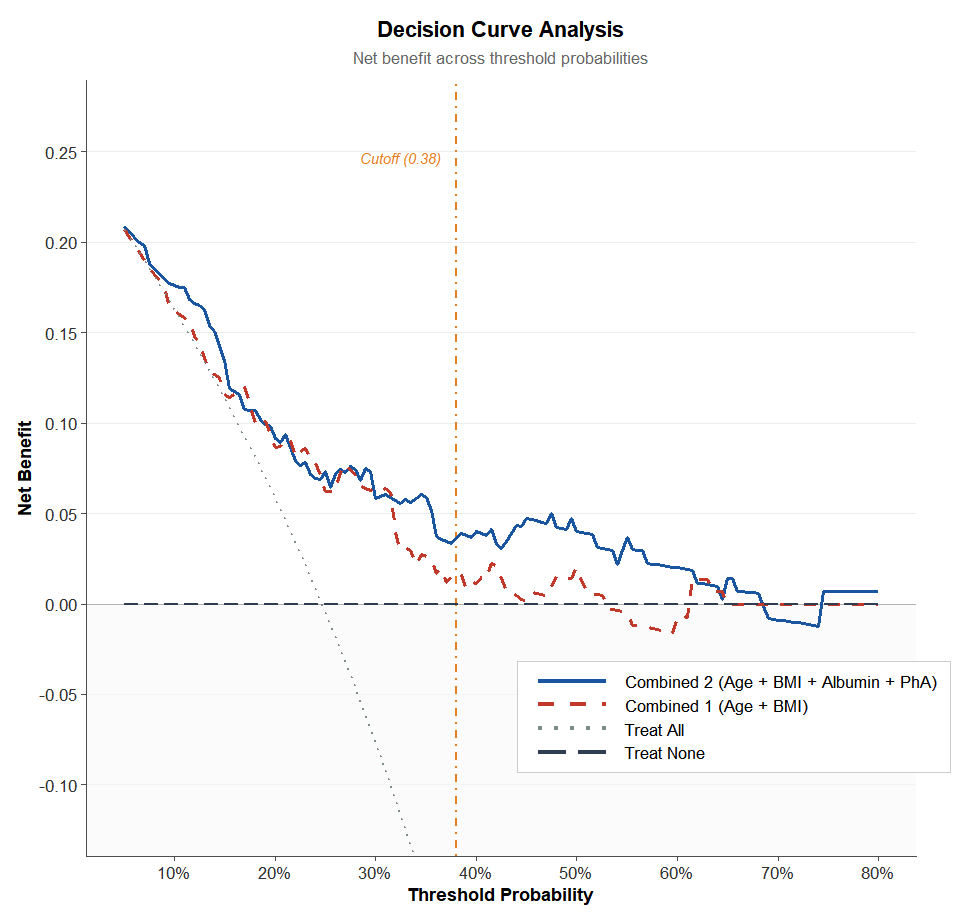

Supplement: Supplementary Figure 1 — Decision curve analysis for the prediction models of low psoas muscle radiodensity (PMD). The y-axis represents net benefit and the x-axis represents the threshold probability. The solid line represents the Combined 2 model (age, BMI, albumin, and phase angle); the dashed line represents the Combined 1 model (age and BMI); the dotted line represents the “treat all” strategy; and the dash-dotted line represents the “treat none” strategy (net benefit = 0). Both models demonstrated positive net benefit across a range of threshold probabilities from approximately 0.10–0.50, supporting their clinical utility for risk stratification when CT-based assessment is unavailable. [file Image_1.TIFF]
