# Supplementary material for: A clinical prediction model for low psoas muscle radiodensity in adults with severe obesity: development and internal validation
Source: Front Nutr. 2026 Jul 13;13:1864098. doi: 10.3389/fnut.2026.1864098 (PMC13403797; doi:10.3389/fnut.2026.1864098)
Supplement: Supplementary file 2 [file Table_1.DOCX]

**Supplementary Table 1. Correlations between PMD and clinical variables**

| Variables | Unadjusted | | Adjusted | |
| --- | --- | --- | --- | --- |
|  | *r* or *rho* | p-value | *r* or *rho* | p-value |
| BMI, kg/m^2^ | -0.270^a^ | <0.001 | -0.412 | **<0.001** |
| Body fat, % | -0.225 | 0.006 | -.0321 | **<0.001** |
| PhA, degree (°) | 0.364 | <0.001 | 0.335 | **<0.001** |
| Albumin, g/dL | 0.297 | <0.001 | 0.204 | **0.013** |
| Iron, μg/dL | 0.239 | 0.003 | 0.218 | **0.008** |
| ln(hsCRP) | -0.104 | 0.166 | -0.236 | **0.009** |
| LDH, U/dL | -0.138 | 0.092 | -0.202 | **0.014** |
| T3, ng/dL | 0.287 | <0.001 | 0.230 | **0.005** |
| TSH, μIU/dL | -0.154 | 0.059 | -0.175 | **0.033** |
| Data are presented as correlation coefficients (*r* or *rho*). Values indicated by ª represent Spearman’s rank correlation coefficients, while others represent Pearson’s correlation coefficients. Adjusted values are controlled for age and sex. Abbreviations: PMD, psoas muscle density; BMI, body mass index; PhA, phase angle; ln(hsCRP), natural logarithm of high-sensitivity C-reactive protein; LDH, lactate dehydrogenase; T3, triiodothyronine; TSH, thyroid-stimulating hormone. | | | | |
